# Supplementary material for: ALS2-Related Motor Neuron Diseases: From Symptoms to Molecules
Source: Biology (Basel). 2022 Jan 5;11(1):77. doi: 10.3390/biology11010077 (PMC8773251; doi:10.3390/biology11010077)
Supplement: Supplementary file 1 [file biology-11-00077-s001.zip › supplementary.pdf]

Supporting Information

S.1. ALS2 Known Mutations

S.1.1. IAHSP

Table 1. Mutations discovered in various studies.

Family	Origin	N.patients	Mutation cDNA	Mutation protein	Onset
1	Kuwait (1,2)	3	c.1425_1426delAG	p.E476Gfs*71	11 mo
2	Algeria (3,4)	3	c.3619delA	p.M1207*	1 yr
3	France (3,4)	1	c.1472_1481del	p.V491Gfs*3	1.5yr
4	Italy (3,4)	1	c.2537_2538delAT	p.N846Ifs*13	16 mo
5	Italy (3,4)	1	c.1007_1008delTA	p.I336Tfs*5	1.5 yr
6	Pakistan (5)	1	c.4721delT	p.V1574Afs*44	1.5 yr
7	Buchari Jewish (6)	2	c.2992C > T	p.R998*	14 mo
8	Turkey (7)	2	c.470G > A	p.C157Y	< 1 yr
9	The Netherlands (8)	2	c.2143C > T	p.Q715*	1 yr
10	Hungary (9)	2	[c.1821_1825dup] ; [c.3529G > T]	[p.E609Afs*9]; [p.G1177*]	< 1 yr
11	Germany (10)	1	c.1999-2A > T	p.E724fs*32	1.5 yr
12	Italy (11)	1	c.3836 + 1G > T	p.K1234 fs*3	1 yr
13	Portugal (12)	1	[c.1427_1428del]; [c.145G > A]	[p.G477Afs*19]; [p.G49R]	3 yr
14	Saudi Arabia (13)	2	c.2761C > T	p.R921*	2 yr
15	Turkey (14)	4	c.2351 + 2C > A	Splicing site	1.5 yr
16	Cina (15)	2	[c.2351_ 2351 +17del]; [c.1310_1313del]	[p.G437Vfs*9]; [p.E724Gfs*26]	<1 yr
17	Pakistan (16)	6	c.2998delA	p.I1000*	1-2 yr
18	Pakistan (16)	5	c.194 T > C	p.F65S	1-2 yr
19	Pakistan (17)	2	c.1918C > T	p.R640*	1-2 yr
20	Iran (18)	11	c.1640+1G>A	Splicing Site	<1.5 yr
21	Turkey (19)	1	c.1718C>A	p.A573E	19 mo
22	Turkey (19)	1	c.1044C>G	p.Y348*	15 mo
23	Turkey (19)	2	c.3161T>C	p.L1054P	15 mo
24	Turkey (19)	1	c.4573dupG	p.V1525Gfs*17	16 mo
25	Turkey (19)	1	c.470G>A	p.C157Y	14 mo
26	Turkey (19)	2	c.1471+1G>A	Splicing site	12 mo
27	NA (20)	NA	c.4831C>T	p.R1611W	NA

yr, year(s); mo, months; NA, Not Available.

S1.2. JPLS

Table S2: Mutations discovered in various studies.

Family	Origin	N.patients	Mutation cDNA	Mutation protein	Onset
1	Italy (21)	1	c.1619G>A	p.G540E	2 yr
2	Cyprus (22)	3	c. 2980-2A>G	p.993fs*7	2 yr
3	Yemen (19)	3	c.275_276delAT	p.Y92Cfs*11	17 mo

yr, year(s); mo, months.

S1.3. JALS

Table S3: Mutations discovered in various studies.

Family	Origin	N.patients	Mutation cDNA	Mutation protein	Onset
1	Tunisia (2)	12	c.138delA	p.A46Afs*5	3-10 yr
2	Turkey (23)	1	c.553delA	p.T185Lfs*5	<2 yr
3	Japan (24)	2	c.3565delG	p.V1189Wfs*19	13 mo - 3 yr
4	Italy (25)	2	[c.299 G>T] [c.2580-2A>G]	p.S100I Splicing Site	3-6 yr
5	Bangladesh (26)	2	c.2002T>G	p.G668*	1-2 yr
6	Turkey (26)	1	c.4573dupG	p.V1525Gfs*17	2-3 yr
7	Pakistan (27)	4	c.3512+1G>A	splicing site	1 yr
8	Japan (28)	1	c.575C>T	p.P192L	<1 yr

yr, year(s); mo, months.

S2. IAHSP Clinical Features

S2.1. IAHSP

Table S4: Clinical features of family number 1, 2, 3.

Clinical features	*fam 1*	Pt 1	Pt2	Pt3	*fam 2*	Pt 1	Pt2	Pt3	*fam 3*	Pt 1
Loss of ambulation		2yr	NW	NW		1yr	1yr	1yr		4yr
Upper limb involvement		+	+	+		<7yr	<7yr	<7yr		6yr
Dysphagia		NA	NA	NA		+	+	+		13yr
Dysarhtria		3-7yr	5-6yr	+		13yr	13yr	13yr		4yr
Ocular movements		N	N	N		N	N	N		Abn

yr, year; N, normal; NT, never talking; NW, never walking; +, present; -, abstent; Abn, abnormal; NA, not available. (1,2), (3,4).

Table S5: Clinical features of family number 4, 5, 6, 7.

Clinical features	*fam 4*	Pt 1	*fam 5*	Pt 1	*fam 6*	Pt 1	*fam 7*	Pt 1	Pt2
Loss of ambulation		5yr		4yr		+		NW	NW
Upper limb involvement		10yr		9yr		+		2yr	6yr
Dysphagia		18yr		+		NA		NA	NA
Dysarhtria		10yr		9yr		+		3yr	3yr
Ocular movements		Abn		Abn		NA		NA	NA

yr, year; NW, never walking; +, present; Abn, abnormal; NA, not available. (3,4), (5), (6).

Table S6: Clinical features of family number 8, 9, 10.

Clinical features	*fam 8*	Pt 1	Pt2	*fam 9*	Pt 1	Pt2	*fam 10*	Pt 1	Pt 2
Loss of ambulation		NW	NW		NW	NW		4yr	NW
Upper limb involvement		12yr	10yr		<4yr	+		6yr	-
Dysphagia		-	+		5yr	4yr		13yr	-
Dysarhtria		+	+		5yr	4yr		4yr	5yr
Ocular movements		NA	NA		NA	NA		Abn	NA

yr, yearl; NW, never walking; +, present; -, abstent; Abn, abnormal; NA, not available. (7), (8), (9).

Table S7 Clinical features of family number 11, 12, 13, 14.

Clinical features	*fam 11*	**Pt 1**	*fam 12*	**Pt 1**	*fam 13*	**Pt 1**	*fam 14*	**Pt 1**	**Pt 2**
Loss of ambulation		NW		NW		NW		4yr	4yr
Upper limb involvement		<7yr		8yr		6yr		+	NA
Dysphagia		NA		8yr		14yr		<4yr	4yr
Dysarhtria		<7yr		8yr		8yr		+	4yr
Ocular movements		N		N		Abn		NA	NA

yr, year; N, normal; NW, never walking; +, present; Abn, abnormal; NA, not available. (10), (11), (12), (13).

Table S8 Clinical features of family number 15, 16.

Clinical features	*fam 15*	**Pt 1**	**Pt2**	**Pt3**	**Pt4**	*fam 16*	**Pt 1**	**Pt2**
Loss of ambulation		+	NW	NW	NW		NW	NW
Upper limb involvement		+	+	+	+		4yr	+
Dysphagia		+	+	+	+		5yr	+
Dysarhtria		+	NT	+	+		5yr	+
Ocular movements		NA	NA	N	N		NA	NA

yr, year; N, normal; NT, never talking; NW, never walking; +, present; NA, not available. (14), (15).

Table S9 Clinical features of family number 17.

Clinical features	*fam 17*	**Pt 1**	**Pt2**	**Pt3**	**Pt4**	**Pt5**	**Pt6**
Loss of ambulation		NW	NW	NW	NW	NW	NW
Upper limb involvement		+	+	+	+	+	+
Dysphagia		NA	NA	NA	NA	NA	NA
Dysarhtria		+	+	+	+	+	+
Ocular movements		NA	NA	NA	NA	NA	NA

N, normal; NW, never walking; +, present; NA, not available. (16).

Table S10 Clinical features of family number 18, 19.

Clinical features	*fam 18*	**Pt 1**	**Pt2**	**Pt3**	**Pt4**	**Pt5**	*fam 19*	**Pt 1**	**Pt2**
Loss of ambulation		NW	NW	NW	NW	NW		NW	NW
Upper limb involvement		+	+	+	+	+		-	-
Dysphagia		5yr	5yr	5yr	5yr	5yr		-	-
Dysarhtria		5yr	5yr	5yr	5yr	5yr		+	+
Ocular movements		N	N	N	N	N		N	N

yr, year; N, normal; NW, never walking; +, present; -, abstent. (16), (17).

Table S11 Clinical features of family number 20.

Clinical features	*fam 20*	**Pt 1**	**Pt2**	**Pt3**	**Pt4**	**Pt5**	**Pt6**	**Pt7**	**Pt8**	**Pt9**	**Pt10**	**Pt11**
Loss of ambulation		NW	NW	NW	1yr	NW	NW	NW	NW	NW	NW	NW
Upper limb involvement		+	+	+	+	+	+	+	+	+	+	+
Dysphagia		-	-	7yr	8yr	<2yr	6yr	6yr	6yr	6yr	6yr	6yr
Dysarhtria		+	+	NT	7yr	NT	6yr	+	+	+	+	+
Ocular movements		N	N	N	N	N	N	N	N	N	N	N

yr, year; N, normal; NT, never talking; NW, never walking; +, present; -, abstent. (18)

Table S12 Clinical features of family number 21, 22, 23, 24.

Clinical features	*fam 21*	Pt 1	*fam 22*	Pt 1	*Fam 23*	Pt1	Pt 1	*fam 24*	Pt 1
Loss of ambulation		-		-		-	8yr		NW
Upper limb involvement		-		-		+	-		+
Dysphagia		-		-		-	-		+
Dysarhtria		6yr		9yr		+	+		+
Ocular movements		NA		NA		NA	NA		+

yr, year; NW, never walking; +, present; -, abstent; NA, not available. (19)

Table S13 Clinical features of family number 25, 26, 27.

Clinical features	*fam 25*	Pt 1	*fam 26*	Pt1	Pt 2
Loss of ambulation		-		NW	NW
Upper limb involvement		-		NA	NA
Dysphagia		+		NA	NA
Dysarhtria		+		+	+
Ocular movements		NA		NA	NA

yr, year; NW, never walking; +, present; -, abstent; NA, not available. (19), (12)

S2.2. JPLS

Table S14 Clinical features of family number 1, 2, 3.

Clinical features	fam 1	Pt1	fam 2	Pt 1	Pt2	Pt3	fam 3	Pt1	Pt2	Pt3
Loss of ambulation		19 yr		50 yr	2yr	-		10yr	3yr	NW
Upper limb involvement		<21yr		+	+	+		+	+	+
EMG		Abn		Abn	Abn	Abn		NA	NA	NA
Dysarthria		6 yr		NA	NA	NA		+	+	+
Ocular movements		<21yr		3 yr	2yr	2yr		NA	NA	NA

yr, year; NW, never walking; +, present; -, absent; Abn, abnormal; NA, not available. (21), (19), (22)

S2.3. JALS

Table S15 Clinical features of family number 1.

Clinical features	*fam 1*	Pt 1	Pt2	Pt3	Pt4	Pt5	Pt6	Pt7	Pt8	Pt9	Pt10	Pt11	Pt12
Loss of ambulation		*NA*	*NA*	*NA*	*NA*	*NA*	*NA*	*NA*	*NA*	*NA*	*NA*	*NA*	*NA*
Upper limb involvement		*NA*	*NA*	*NA*	*NA*	*NA*	*NA*	*NA*	*NA*	*NA*	*NA*	*NA*	*NA*
EMG		*NA*	*NA*	*NA*	*NA*	*NA*	*NA*	*NA*	*NA*	*NA*	*NA*	*NA*	*NA*
Dysarthria		*NA*	*NA*	*NA*	*NA*	*NA*	*NA*	*NA*	*NA*	*NA*	*NA*	*NA*	*NA*
Ocular movements		*10yr*	*6yr*	+	*6yr*	*9yr*	*6yr*	*6yr*	+	+	+	+	+

yr, year; +, present; NA, not available. (2)

Table S16 Clinical features of family number 2, 3, 4.

Clinical features	*fam 2*	Pt 1	*fam 3*	Pt1	Pt 2	*fam 4*	Pt1	Pt 2
Loss of ambulation		16yr		-	-		NA	NA
Upper limb involvement		12yr		NA	NA		+	+
EMG		Abn		N	N		NA	NA
Dysarthria		18yr		11yr	11yr		7yr	7yr
Ocular movements		15yr		11yr	11yr		+	+

yr, year; +, present; -, absent; Abn, abnormal; NA, not available. (23), (24), (25)

Table S17 Clinical features of family number 5, 6.

Clinical features	*fam 5*	**Pt1**	**Pt 2**	*fam 6*	**Pt 2**
Loss of ambulation		+	+		8yr
Upper limb involvement		+	+		+
EMG		NA	NA		NA
Dysarthria		+	+		4yr
Ocular movements		+	+		+

yr, year; +, present; NA, not available. (26)

Table S18 Clinical features of family number 7, 8.

Clinical features	*fam 7*	**Pt1**	**Pt2**	**Pt3**	**Pt 4**	*fam 8*	**Pt1**
Loss of ambulation		5yr	NW	NA	NA		NA
Upper limb involvement		+	+	NA	NA		NA
EMG		NA	NA	NA	NA		NA
Dysarthria		+	7yr	NA	NA		NA
Ocular movements		+	+	NA	NA		NA

yr, year; +, present; NA, not available; NW, never walking. (27), (28)

Bibliography

1. Lerman-Sagie T, Filiano J, Warwick Smith D, Korson M. Infantile Onset of Hereditary Ascending Spastic Paralysis With Bulbar Involvement. J Child Neurol. 1996 Jan;11(1):54–7.
2. Hadano S, Hand CK, Osuga H, Yanagisawa Y, Otomo A, Devon RS, et al. A gene encoding a putative GTPase regulator is mutated in familial amyotrophic lateral sclerosis 2. Nat Genet [Internet]. 2001 Oct;29(2):166–73. Available from: http://www.nature.com/articles/ng1001-166
3. Lesca G, Eymard–Pierre E, Santorelli FM, Cusmai R, Di Capua M, Valente EM, et al. Infantile ascending hereditary spastic paralysis (IAHSP). Neurology [Internet]. 2003 Feb 25;60(4):674–82. Available from: http://www.neurology.org/lookup/doi/10.1212/01.WNL.0000048207.28790.25
4. Eymard-Pierre E, Lesca G, Dollet S, Santorelli FM, di Capua M, Bertini E, et al. Infantile-Onset Ascending Hereditary Spastic Paralysis Is Associated with Mutations in the Alsin Gene. Am J Hum Genet. 2002 Sep;71(3):518–27.
5. Gros-Louis F, Meijer IA, Hand CK, Dubé M-P, MacGregor DL, Seni M-H, et al. An ALS2 gene mutation causes hereditary spastic paraplegia in a Pakistani kindred. Ann Neurol. 2003 Jan;53(1):144–5.
6. Devon R, Helm J, Rouleau G, Leitner Y, Lerman-Sagie T, Lev D, et al. The first nonsense mutation in alsin results in a homogeneous phenotype of infantile-onset ascending spastic paralysis with bulbar involvement in two siblings. Clin Genet. 2003 Sep;64(3):210–5.
7. Eymard-Pierre E, Yamanaka K, Haeussler M, Kress W, Gauthier-Barichard F, Combes P, et al. Novel missense mutation in ALS2 gene results in infantile ascending hereditary spastic paralysis. Ann Neurol. 2006 Jun;59(6):976–80.
8. Verschuuren-Bemelmans CC, Winter P, Sival DA, Elting J-W, Brouwer OF, Müller U. Novel homozygous ALS2 nonsense mutation (p.Gln715X) in sibs with infantile-onset ascending

spastic paralysis: the first cases from northwestern Europe. Eur J Hum Genet. 2008 Nov;16(11):1407–11.

9. Sztriha L, Panzeri C, Kálmánchey R, Szabó N, Endreffy E, Túri S, et al. First case of compound heterozygosity in ALS2 gene in infantile-onset ascending spastic paralysis with bulbar involvement. Clin Genet. 2008 Apr;73(6):591–3.

10. Herzfeld T, Wolf N, Winter P, Hackstein H, Vater D, Müller U. Maternal uniparental heterodisomy with partial isodisomy of a chromosome 2 carrying a splice acceptor site mutation (IVS9–2A>T) in ALS2 causes infantile-onset ascending spastic paralysis (IAHSP). Neurogenetics [Internet]. 2009 Feb 23;10(1):59. Available from: http://link.springer.com/10.1007/s10048-008-0148-y

11. Racis L, Tessa A, Pugliatti M, Storti E, Agnetti V, Santorelli FM. Infantile-onset ascending hereditary spastic paralysis: A case report and brief literature review. Eur J Paediatr Neurol [Internet]. 2014 Mar;18(2):235–9. Available from: https://linkinghub.elsevier.com/retrieve/pii/S1090379813001463

12. Flor-de-Lima F, Sampaio M, Nahavandi N, Fernandes S, Leão M. Alsin Related Disorders: Literature Review and Case Study with Novel Mutations. Case Rep Genet [Internet]. 2014;2014:1–5. Available from: http://www.hindawi.com/journals/crig/2014/691515/

13. Wakil SM, Ramzan K, Abuthuraya R, Hagos S, Al-Dossari H, Al-Omar R, et al. Infantile-onset ascending hereditary spastic paraplegia with bulbar involvement due to the novel ALS2 mutation c.2761C > T. Gene. 2014 Feb;536(1):217–20.

14. Koçak Eker H, Ünlü SE, Al-Salmi F, Crosby AH. A novel homozygous mutation in ALS2 gene in four siblings with infantile-onset ascending hereditary spastic paralysis. Eur J Med Genet. 2014 May;57(6):275–8.

15. Xie F, Cen Z, Xiao J, Luo W. Novel compound heterozygous ALS2 mutations in two Chinese siblings with infantile ascending hereditary spastic paralysis. Neurol Sci. 2015 Jul;36(7):1279–80.

16. Daud S, Kakar N, Goebel I, Hashmi AS, Yaqub T, Nürnberg G, et al. Identification of two novel ALS2 mutations in infantile-onset ascending hereditary spastic paraplegia. Amyotroph Lateral Scler Front Degener [Internet]. 2016 May 18;17(3–4):260–5. Available from: https://www.tandfonline.com/doi/full/10.3109/21678421.2015.1125501

17. Tariq H, Mukhtar S, Naz S. A novel mutation in ALS2 associated with severe and progressive infantile onset of spastic paralysis. J Neurogenet. 2017 Apr;31(1–2):26–9.

18. Helal M, Mazaheri N, Shalbafan B, Malamiri RA, Dilaver N, Buchert R, et al. Clinical presentation and natural history of infantile-onset ascending spastic paralysis from three families with an ALS2 founder variant. Neurol Sci. 2018;39(11):1917–25.

19. Sprute R, Jergas H, Ölmez A, Alawbathani S, Karasoy H, Salimi Dafsari H, et al. Genotype–phenotype correlation in seven motor neuron disease families with novel <scp> *ALS2* </scp> mutations. Am J Med Genet Part A. 2020 Nov;ajmg.a.61951.

20. Sato K, Otomo A, Ueda MT, Hiratsuka Y, Suzuki-Utsunomiya K, Sugiyama J, et al. Altered oligomeric states in pathogenic ALS2 variants associated with juvenile motor neuron diseases cause loss of ALS2-mediated endosomal function. J Biol Chem [Internet]. 2018 Nov 2;293(44):17135–53. Available from: http://www.jbc.org/lookup/doi/10.1074/jbc.RA118.003849

21. Panzeri C. The first ALS2 missense mutation associated with JPLS reveals new aspects of alsin biological function. Brain. 2006 Jul;129(7):1710–9.

22. Mintchev N, Zamba-Papanicolaou E, Kleopa KA, Christodoulou K. A novel ALS2 splice-site mutation in a Cypriot juvenile-onset primary lateral sclerosis family. Neurology. 2009 Jan;72(1):28–32.

23. Kress JA, Kühnlein P, Winter P, Ludolph AC, Kassubek J, Müller U, et al. Novel mutation in theALS2 gene in juvenile amyotrophic lateral sclerosis. Ann Neurol. 2005 Nov;58(5):800–3.

24. Shirakawa K, Suzuki H, Ito M, Kono S, Uchiyama T, Ohashi T, et al. NOVEL COMPOUND HETEROZYGOUS ALS2 MUTATIONS CAUSE JUVENILE AMYOTROPHIC LATERAL SCLEROSIS IN JAPAN. Neurology. 2009 Dec;73(24):2124–6.

25. Luigetti M, Lattante S, Conte A, Romano A, Zollino M, Marangi G, et al. A novel compound heterozygous ALS2 mutation in two Italian siblings with juvenile amyotrophic lateral sclerosis. Amyotroph Lateral Scler Front Degener [Internet]. 2013 Sep 3;14(5–6):470–2. Available from: http://www.tandfonline.com/doi/full/10.3109/21678421.2012.756036

26. Sheerin U-M, Schneider SA, Carr L, Deuschl G, Hopfner F, Stamelou M, et al. ALS2 mutations: Juvenile amyotrophic lateral sclerosis and generalized dystonia. Neurology. 2014 Mar;82(12):1065–7.

27. Siddiqi S, Foo JN, Vu A, Azim S, Silver DL, Mansoor A, et al. A Novel Splice-Site Mutation in ALS2 Establishes the Diagnosis of Juvenile Amyotrophic Lateral Sclerosis in a Family with Early Onset Anarthria and Generalized Dystonias. Raoul C, editor. PLoS One. 2014 Dec;9(12):e113258.

28. Nishiyama A, Niihori T, Warita H, Izumi R, Akiyama T, Kato M, et al. Comprehensive targeted next-generation sequencing in Japanese familial amyotrophic lateral sclerosis. Neurobiol Aging. 2017 May;53:194.e1-194.e8.
